# Influence of Coffee Oil Epoxide as a Bio-Based Plasticizer on the Thermal, Mechanical, and Barrier Performance of PHBV/Natural Rubber Blends

**DOI:** 10.3390/polym18020240

**Published:** 2026-01-16

**Authors:** Rinky Ghosh, Xiaoying Zhao, Marie Genevieve Boushelle, Yael Vodovotz

**Affiliations:** 1 Department of Food Science and Technology, The Ohio State University, 2015 Fyffe Road, Columbus, OH 43210, USA; ghosh.245@osu.edu; 2 School of Light Industry Science and Engineering, Beijing Technology and Business University, No. 33 Fucheng Road, Beijing 100048, China; 3 Department of Chemistry and Biochemistry, The Ohio State University, 200 CBEC Building 151 W. Woodruff Ave. Columbus, OH 43210, USA

**Keywords:** PHBV, natural rubber, coffee oil epoxide, bio-plasticizer, thermal properties, barrier properties, sustainable packaging

## Abstract

This work evaluated the effect of coffee oil epoxide (COE), produced from coffee waste, on thermal, mechanical, barrier, and exudation resistance properties of poly(3-hydroxybutyrate-co-3-hydroxyvalerate)/natural rubber (PHBV/NR) blends. Building upon previously published 0.3% COE results, this study examined 0.4% and 0.75% concentrations to optimize performance. Thermal analysis revealed that COE incorporation significantly enhanced chain mobility, with glass transition temperature depressions of 6.1 °C and 7.4 °C for 0.4% and 0.75% COE formulations, respectively, compared to unplasticized PHBV/NR blends. Crystallinity decreased from 54.5% (PHBV/NR) to 52.6% and 51.9% with increasing plasticizer concentration, while melting temperatures decreased by 3.9% and 4.9%, confirming improved polymer chain mobility. Mechanical properties demonstrated COE’s plasticizing effectiveness, with tensile strength decreasing by 13.3% (0.4% COE) and 16.2% (0.75% COE) compared to PHBV/NR blends. Young’s modulus similarly decreased by 21.0% and 24.0%, while elongation at break improved slightly with increasing COE content. Barrier properties improved substantially across all concentrations: water vapor transmission rates decreased from 4.05 g/m^2^·h (PHBV/NR) to 1.55 g/m^2^·h (0.3% COE) and 0.67 g/m^2^·h for 0.4% and 0.75% COE, attributed to COE’s hydrophobic nature. SEM morphological analysis confirmed improved phase compatibility at 0.40% COE, with reduced rubber droplet size and homogeneous surface morphology. Exudation testing revealed excellent retention (0.21–0.53 wt% loss over 63 days). Results indicate 0.40% COE as optimal, achieving superior barrier properties while maintaining mechanical performance for sustainable packaging applications.

## 1. Introduction

As packaging manufacturers and researchers seek efficient biodegradable and sustainable packaging solutions, microbial biopolymer poly(3-hydroxybutyrate-co-hydroxyvalerate) (PHBV) has demonstrated complete biodegradability in both marine and soil environments [1,2]. A study by Weng et al. [3] found 100% biodegradability of PHBV under composting conditions and 81% biodegradation in controlled laboratory settings, making it a unique and promising alternative to petroleum-based plastics. PHBV exhibits high crystallinity, resulting in elevated melting temperatures (170–180 °C) and superior barrier properties against water vapor and oxygen [4,5,6]. However, high crystallinity induces brittleness and low elongation at break, while its high production cost further limits commercial viability [7,8]. Another alternative investigated to improve the toughness and flexibility of PHBV is natural rubber (NR), which offers cost-effectiveness, durability, and resilience with excellent elastic properties [9]. Despite these advantages, experimental studies have highlighted processing limitations, particularly poor sheet formability [10].

Furthermore, to enhance flexibility and improve elongation at break, plasticizers are commonly incorporated into PHBV [7]. Various bio-based plasticizers such as glycerol, soybean oil, oxypropylated glycerin, acetyl tributyl citrate (ATBC), polyethylene glycol (PEG), and epoxidized soybean oil have been explored due to their low cost, availability, and renewable origins [11,12,13]. However, certain plasticizers pose health risks, such as endocrine disruption [14]. Consequently, exploring non-toxic alternatives for safe packaging applications is crucial. Research by Requena et al. investigated the use of polyethylene glycol, lauric acid, and stearic acid as plasticizers for PHBV. Their hypothesis was that these plasticizers would reduce intermolecular forces between polymer chains, leading to increased flexibility and modified crystallization behavior [15]. The study found that only PEG effectively improved flexibility at the concentrations tested, but it did not meet packaging requirements.

To address these processing constraints, previous research indicates that coffee oil epoxide (a bio-based plasticizer) addition to PHBV/NR blends creates products with commercialization potential [16]. Coffee oil epoxide (COE), derived from coffee processing waste, has emerged as a promising candidate due to its ability to improve polymer flexibility while contributing to waste valorization [17]. Although epoxidized soybean oil (ESO) has been more extensively studied [18,19], COE presents distinct advantages, as coffee waste represents nearly 90% of post-consumption residues and contains 11–20% oil by weight depending on the extraction method [20,21]. Landfilled coffee waste is environmentally detrimental, releasing caffeine, tannins, polyphenols, and methane, thereby reinforcing the need for sustainable applications of this resource [22,23].

Previous research by our group demonstrated that 0.3% COE addition to PHBV/NR blends improved processability and barrier properties, creating products with commercialization potential [10,16]. However, the effects of higher COE concentrations on blend properties remain unexplored. The objective of this study was to systematically examine the effects of varying coffee oil epoxide concentrations (0.40% and 0.75%) on PHBV/NR blend properties, specifically analyzing thermal transitions, mechanical and barrier properties, and plasticizer exudation behavior to optimize formulations for sustainable packaging applications.

## 2. Experimental Section

### 2.1. Materials

Poly(3-hydroxybutyrate-co-3-hydroxyvalerate) (PHBV) granules containing 2 mol% hydroxyvalerate with a molecular weight of 280 kDa were acquired from Tianan Biological Material Co. (Ningbo, China). Trimethylolpropane triacrylate (TMPTA) was purchased from Sigma-Aldrich (St. Louis, MO, USA), while 2,5-Bis(tert-butylperoxy)-2,5-dimethylhexane (Luperox 101XL45, 92%, MW: 290.44) was sourced from Thermo Fisher Scientific, Ridgefield, NJ, USA. Natural rubber (NR) was provided by Midwest Elastomers Inc., Wapakoneta, OH, USA. Coffee oil epoxide (COE) was synthesized according to the procedure outlined by Williamson et al. [17]. Both NR and the peroxide were utilized without further modification. Blends of PHBV/NR/COE with different COE contents (0.40% and 0.75%) were mixed using an internal mixer prior to melt compounding.

### 2.2. Melt Compounding

Prior to processing, PHBV pellets were vacuum dried at 60 °C for 24 h, resulting in a final moisture content of 0.1% in the blends. The optimized blend formulation comprised of 85 wt% PHBV, 15 wt% NR, 0.45 wt% peroxide, 0.63 wt% coagent, and 0.40 and 0.75 wt% COE. Melt compounding was performed using a Leistritz ZSE-27 (Allendale, NJ, USA) co-rotating twin-screw extruder (D = 27 mm, L/D = 40:1) with a reverse temperature profile decreasing from 180 to 160 °C. The screw speed and throughput were maintained at 64 rpm and ~10 kg/h respectively, with screw configuration optimized to minimize thermal degradation during processing. The extrudate was immediately pelletized using a Scheer Bay pelletizer at a feed roller speed of 2–3 rpm [16].

Sheet extrusion was conducted by reprocessing the compounded pellets using a single screw extruder equipped with a coat-hanger die Davis Standard Extruder (DS20, 24:1 L/D, Pawcatuck, CT, USA) (Table 1). The extruded sheets were passed through a water-cooled roll stand maintained at ambient temperature (~22–25 °C) to prevent surface adhesion and ensure dimensional stability. Although the cooling rate was not directly quantified, identical cooling conditions and take-off speed were maintained for all formulations to ensure comparable thermal histories. The resulting sheets exhibited an average thickness of approximately 0.42 mm, with variations within ±0.03 mm across all samples. Processing parameters remained constant to maintain experimental reproducibility. The blends containing PHBV/NR with coagent, and peroxide were designated as PHBV/NR, while those incorporating COE were denoted as PHBV/NR/COE.

### 2.3. Characterization of PHBV and Its Blends

#### 2.3.1. Thermal Transition Measurements-DSC Analysis

The thermal behavior of PHBV/NR/COE polymeric sheets containing 0.40 and 0.75% COE was investigated using a Discovery DSC 2500 instrument (TA Instruments, New Castle, DE, USA). Prior to testing, all specimens were vacuum dried at 60 °C for 24 h to remove residual moisture. Samples weighing 5–10 mg were accurately weighed and hermetically sealed in T_zero_ aluminum pans. Samples were subjected to the following temperature program: initial heating from ambient temperature to 200 °C at a heating rate of 10 °C/min, isothermal holding at 200 °C for 5 min to ensure complete melting and elimination of thermal history, cooling to −85 °C at 10 °C/min, equilibration at −85 °C for 5 min, followed by a second heating scan to 200 °C at 10 °C/min.

Melting temperatures (T_m_) were determined from the peak maxima of the endothermic transitions observed during the second heating scan. The degree of crystallinity (X_c_) was calculated according to the Equation (1):

(1) $$X_{c}=\frac{\Delta H_{m}}{\Delta {H{}^{\circ}}_{m}}\times 100\%$$

where ΔH_m_ represents the measured enthalpy of fusion obtained by integrating the area under the melting endotherm using TRIOS Software (v4.1.1.33073), and ΔH°_m_ is the theoretical enthalpy of fusion for 100% crystalline PHBV (146 J/g) as reported in literature [24]. All measurements were performed in triplicate to ensure reproducibility of the thermal data.

#### 2.3.2. Mechanical Testing

The tensile properties of the polymer films were characterized in accordance with ASTM D882-18 standard [25]. Test specimens with dimensions of 100 mm × 10 mm × 0.42 mm were prepared from each formulation and subjected to uniaxial tensile testing using a grip separation distance of 50 mm. Mechanical testing was conducted using an Instron Universal Testing Machine (Model 34TM-50, Instron Corp., Norwood, MA, USA) equipped with Bluehill software (v.2.17). All tests were performed at ambient temperature conditions with a crosshead speed of 10 mm/min. For each sample composition, ten replicate specimens were tested to ensure statistical reliability. Data analysis was performed using JMP Pro 16.0 statistical software (SAS Institute, Cary, NC, USA). Significant differences between PHBV/NR/COE formulations containing 0.40% and 0.75% COE were assessed using one-way analysis of variance (ANOVA) followed by Tukey’s honestly significant difference (HSD) post-hoc test, with statistical significance established at *p* < 0.05.

#### 2.3.3. Barrier Properties

Water vapor transmission rate (WVTR) measurements were performed on PHBV/NR blend films containing 0.4%, and 0.75% COE using a dynamic vapor sorption analyzer (Surface Measurement Systems Ltd., Allentown, PA, USA) according to ASTM E96/E96M-16 standard [26] protocol. Test conditions were maintained at 40 °C for 24 h duration. Circular film specimens with approximately 7 mm diameter and ~0.42 mm thickness was prepared from each blend composition and mounted onto Payne permeation cells featuring a 15.54 mm^2^ exposed area. The experimental setup established a humidity gradient across the film samples, with 0% relative humidity (RH) maintained on the desiccant side using a drying agent, while the opposite side was exposed to 90% RH environment.

Water vapor permeability was determined by monitoring the mass change of the desiccant over the testing period. For statistical reliability, triplicate measurements were conducted for each COE concentration and the unplasticized PHBV/NR control, and average values with standard deviations were calculated.

Water vapor transmission rate (g/m^2^·h) was calculated using the given ASTM formula, Equation (2):

(2) $$WVTR=\frac{Slope\;of\;the\;curve\;(G)}{Time\;\left(t\right)\times planar\;area\;of\;sheet}$$

#### 2.3.4. Exudation and Volatility Resistance Testing

Plasticizer migration from the PHBV and its blended sheets was assessed using an accelerated exudation test [27]. Samples were placed on aluminum trays covered with filter papers and positioned in a shaker within an Isotemp Fisherbrand Oven (Thermo Fisher Scientific, Pittsburgh, PA, USA) maintained at 50 °C for 63 days. Sample masses were measured weekly, with filter papers being replaced after each measurement. Mass loss percentage was calculated using the following Equation (3):

(3) $$Exudation\;\left(\%\right)=\frac{M_{i}\;-\;M_{f}}{M_{i}}\times 100$$

where M_i_ represents the initial mass of the sample and M_f_ denotes the mass at each time point. Measurements were performed in triplicate to ensure reproducibility of results.

Volatility resistance was assessed using the activated carbon method following ISO 176:2005 standard [28]. Test specimens were positioned at the base of sealed metal containers with 120 cm^3^ of activated carbon uniformly distributed over the sample surfaces. The sealed containers were transferred to a convection oven (BINDER Inc., Bohemia, NY, USA) and maintained at 70 ± 1 °C for 24 h. Following thermal exposure, containers were cooled to ambient temperature, and specimens were carefully brushed to remove adhering carbon particles before reweighing. Weight changes were recorded to determine volatile loss. Triplicate measurements were conducted for each COE formulation to ensure statistical reliability.

#### 2.3.5. Surface Morphology Analysis

Surface morphology of PHBV/NR blends plasticized with 0.3%, 0.40%, and 0.75% COE was examined using scanning electron microscopy (Apreo II, Thermo Fisher Scientific, USA). The instrument operates at voltages ranging from 200 V to 30 kV with beam currents from 1 pA to 400 nA. Prior to imaging, vacuum-dried samples were iridium-coated to a thickness of 10 nm using a Leica ACE600 Sputter Coater (Wetzlar, Germany) to minimize charging effects during analysis. Imaging was performed utilizing Everhart-Thornley Detector (ETD) and T2 detectors for topographical analysis. Particle size measurements from SEM micrographs were analyzed using ImageJ software (version 1.54k, National Institutes of Health, Bethesda, MD, USA) to determine mean diameter distributions.

## 3. Results and Discussion

All formulations were based on the optimized blend composition of 85 wt% PHBV and 15 wt% NR with 0.45 wt% peroxide and 0.63 wt% coagent, with coffee oil epoxide (COE) added at 0.40 and 0.75 wt% of the total formulation weight.

### 3.1. Effect of Plasticizer on Glass Transition and Melting Behaviors

**Figure 1:**
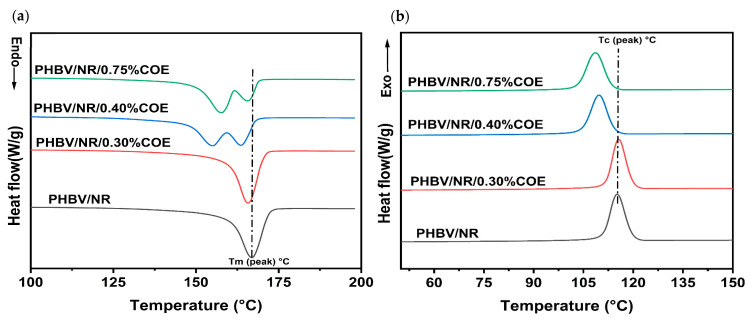
DSC (**a**) heating, (**b**) cooling cycles of PHBV/NR blends with various % of coffee oil epoxide.

The DSC thermograms of PHBV/NR/COE blends reveal the significant influence of coffee oil epoxide (COE) on the melting and cooling cycles of PHBV/NR (85:15) blends (Table 2). The incorporation of COE systematically decreased the glass transition temperature (T_g_) of PHBV, showing depressions of 6.1 °C and 7.4 °C for the 0.4% and 0.75% COE formulations, respectively, compared to PHBV/NR blends. The melting enthalpy (ΔH_m_) decreased progressively with increasing COE content, accompanied by corresponding reductions in crystallinity from 54.5% (PHBV/NR) to 52.6% and 51.9% for the 0.40% and 0.75% COE blends, respectively. Similarly, the peak melting temperatures of plasticized blends decreased by 3.9% and 4.9% for the two formulations. This change was attributed to weakened intermolecular forces between adjacent polymeric chains due to low-molecular-weight plasticizer molecule intercalation, consistent with findings by Slongo et al. [19] for PHBV plasticized with epoxidized vegetable oils. Furthermore, the DSC analysis revealed the presence of double melting peaks in both the 0.40% and 0.75% COE formulations (Figure 1a). The melting peak appeared at a lower temperature with a shoulder visible at higher temperatures, indicating heterogeneous crystalline domains with different lamellar thicknesses [29]. This phenomenon arises from compositional heterogeneity and non-uniform chain interactions introduced by COE addition [30]. The formation of different crystalline phases, as documented in literature for PHBV systems, contributes to this complex thermal behavior [19]. During the cooling cycle, the melt crystallization temperature shifted to lower values with increasing COE, and the enthalpy of crystallization decreased by 3.5% and 4.8% for the 0.40% and 0.75% formulations, respectively (Figure 1b). This further supports the disruption of crystal nucleation and growth by plasticizer incorporation [31]. This plasticizer-induced crystallinity reduction is likely influencing the observed Young’s modulus decrease (see discussion below). The concurrent reduction in both glass transition and melting temperatures establishes COE as an effective processing aid, expanding the PHBV processing window by enabling lower temperature processing while avoiding thermal degradation [32].

### 3.2. Effect of COE on Mechanical Properties of PHBV/NR Blends

The mechanical properties of PHBV/NR blends with varying COE content are presented in Table 3. Pure PHBV data is provided for reference; all subsequent comparisons use PHBV/NR as the baseline control. Neat PHBV demonstrates characteristic brittle behavior with high young’s modulus and minimal elongation at break. Previous investigations compared PHBV, PHBV/NR and PHBV/NR/0.30% COE formulations; however, low plasticizer concentrations exhibited negligible influence on elongation properties. This study examines higher plasticizer concentrations versus unplasticized PHBV/NR blends. Tensile strength decreased progressively with increasing COE content. The PHBV/NR blend (24.33 ± 1.15 MPa) decreased by 13.3% with 0.40% COE and 16.2% with 0.75% COE. This reduction reflects plasticizer-induced disruption of intermolecular forces between polymer chains. Similar results were observed for PHBV and Poly(3-hydroxybutyrate) (PHB) plasticized with poly(ethylene glycol) (PEG), acetyl tributyl citrate (ATBC), epoxidized soybean oil, and tributyrin [18,33]. Young’s modulus followed similar decreasing patterns. The PHBV/NR control (1421.28 ± 110.10 MPa) decreased by 21.0% and 24.0% for 0.40% and 0.75% COE additions, respectively, confirming COE’s plasticizing effect through increased chain mobility. Consistent with the reduction in stiffness, the elongation at break exhibited an opposite trend, increasing with higher COE concentrations. Although not all differences were statistically significant, the elongation at break showed a general increasing trend with higher COE concentrations, rising from the PHBV/NR control (2.89 ± 0.35%) by approximately 15.9% and 37.4% for the 0.40% and 0.75% COE formulations, respectively. The 0.75% COE blend exhibited the highest elongation at break (3.97 ± 1.12%), indicating improved flexibility of the blends. These findings corroborate established literature trends. Seydibeyoğlu et al. [34] reported that 10 wt% epoxidized linseed (ELO) and soybean oils (ESBO) increased elongation while reducing tensile strength and Young’s modulus. Similarly, the incorporation of epoxidized palm oil (EPO) was shown to reduce Young’s modulus from 630 MPa to 580 MPa while enhancing chain mobility in PHBV/PCL blend systems [35]. Additionally, Silverajah et al. [36] demonstrated that 5 wt% epoxidized palm oil enhanced PLA elongation from 6.3% to 130%, confirming the plasticizing efficacy of epoxidized compounds in biobased, biodegradable polymers.

### 3.3. Effect of COE Content on Barrier Properties of PHBV/NR Blends

**Figure 2:**
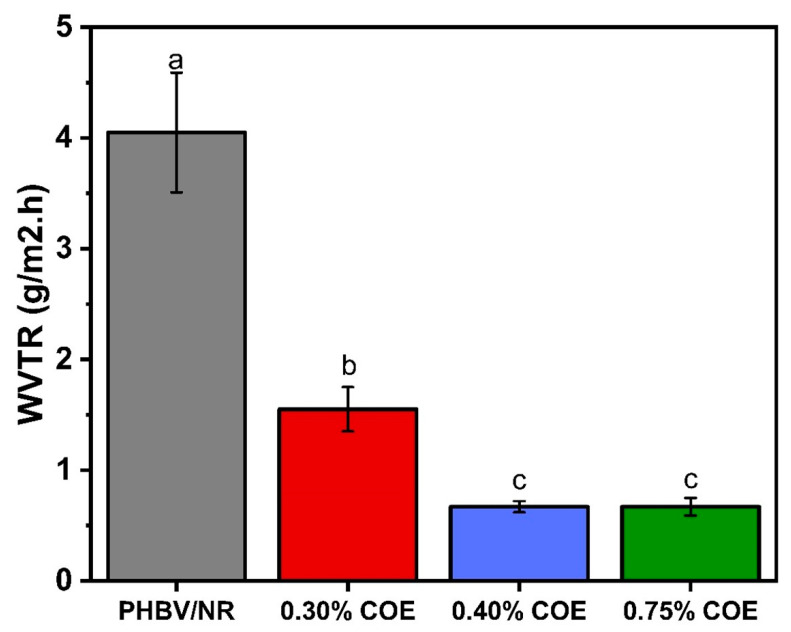
WVTR of PHBV/NR/COE blends at different plasticizer loadings, different letters indicate statistically significant differences between groups (*p* < 0.05).

The addition of COE markedly affected the water vapor transmission rate of PHBV/NR blends, as evidenced in Figure 2. The initial WVTR of the control sheets was 4.05 g/m^2^·h, which was subsequently reduced to 1.55 g/m^2^·h upon the inclusion of 0.30% COE. Further decreases were observed with 0.40% and 0.75% COE, yielding the lowest values of 0.67 ± 0.05 and 0.67 ± 0.08 g/m^2^·h, respectively. This substantial reduction in WVTR is attributable to the hydrophobic nature of COE, a characteristic previously documented by our research group [16]. This hydrophobicity enhances barrier properties by minimizing water-polymer interactions and creating a more tortuous diffusion pathway for water molecules. The lack of a significant difference in WVTR between the 0.4% and 0.75% COE formulations indicates that the polymer matrix reached its saturation point, beyond which additional COE could not effectively impede vapor transport. Similar saturation phenomena have been reported in PHBV films plasticized with various agents, including polypropylene glycol, glycerol, castor oil, and PEG (Jost et al. [18]), as well as in PHBH modified with low-molecular-weight PEG (Farrag et al. [37]). Consequently, these findings suggest that 0.4% COE represents the optimal concentration for barrier enhancement in PHBV/NR/COE blends, concurrently reducing crystallinity, modifying microstructural organization, and minimizing moisture permeation through the films via plasticization and hydrophobic modification.

### 3.4. Effect of COE on Exudation and Volatility Resistance of PHBV/NR Blends

**Figure 3:**
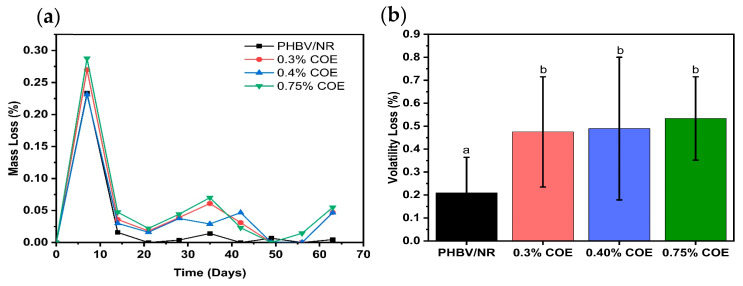
Exudation and volatility resistance of PHBV/NR blends with varying COE content: (**a**) mass loss over time during 63-day exudation testing, (**b**) volatility loss after 24 h at 70 °C, different letters indicate statistically significant differences between groups (*p* < 0.05).

Exudation testing over 63 days revealed minimal cumulative weight loss across all formulations, demonstrating stable COE retention within the polymer matrix (Figure 3a). The low exudation behavior is attributed to hydrogen bonding and dipole-dipole interactions between COE’s polar ester/oxirane groups and PHBV’s carbonyl groups [38]. An initial mass loss peak (0.23–0.29 wt%) occurred during week 1, caused by rapid elimination of water and low-molecular-weight species combined with temperature-induced relaxation of PHBV amorphous regions [27]. Beyond this point, mass loss values sharply decreased, stabilizing during the final three weeks (days 49–63). This plateau indicates depletion of the mobile plasticizer fraction and attainment of a quasi-equilibrium state within the polymer network. Minor fluctuations at intermediate timepoints (day 35 for 0.75% COE; day 42 for PHBV/NR) reflect localized structural reorganizations within the heterogeneous blend matrix. After this period, the system tends toward equilibrium, and the migration rate slows, consistent with the stabilization observed in the final weeks. Similar observations were reported for PHBV plasticized with tributyrin [27]. These results confirm COE’s effectiveness as a durable bio-based plasticizer through sustained molecular interactions with the PHBV matrix.

Volatility resistance measurements showed mass loss increasing from 0.21 ± 0.15% (unplasticized PHBV/NR) to 0.49 ± 0.31% (0.40% COE) and 0.53 ± 0.18% (0.75% COE). At 0.75% COE, there is a slight but not remarkable decrease in volatility resistance with increasing coffee oil epoxide content (Figure 3b). However, the observed volatility losses (0.21–0.53 wt%) are substantially lower than literature values for PVC films plasticized with epoxidized isobutyl esters and epoxidized soybean oil (2–11 wt% loss) [39]. This trend confirms that PHBV/NR blended sheets maintain good volatility resistance in the presence of coffee oil epoxide, demonstrating the blend’s suitability for elevated temperature processing conditions such as extrusion and injection molding, as well as thermally demanding applications.

### 3.5. Effect of COE on Surface Morphology of PHBV/NR Blends

**Figure 4:**
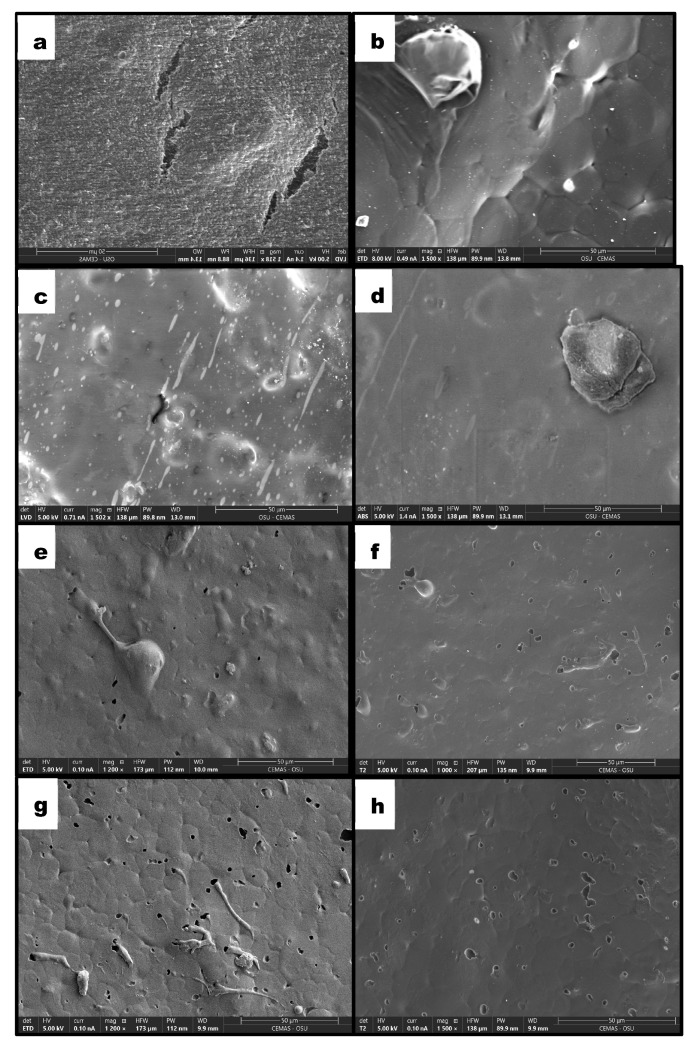
Surface morphology of PHBV/NR blends with varying COE content: (**a**) PHBV, (**b**,**d**) PHBV/NR, (**c**) PHBV/NR/0.3% COE, (**e**,**f**) PHBV/NR/0.40% COE, (**g**,**h**) PHBV/NR/0.75% COE.

Based on previous studies, neat PHBV exhibits a smooth surface with microcracks, consistent with its highly oriented semicrystalline structure and inherent brittleness (Figure 4a) [16]. Incorporation of NR results in phase-separated morphology, evidenced by large rubber domains (~29.79 μm), confirming the immiscible nature of the PHBV/NR binary blend (Figure 4b,d). The addition of 0.3 wt% COE was previously shown to suppress the formation of distinct NR microdroplets, leading to more homogeneous surface morphology [16]. This behavior was attributed to enhanced compatibility arising from coalescence and interaction of COE and NR within the PHBV matrix, promoting improved dispersion and formation of an interconnected polymeric network (Figure 4c).

Upon increasing COE content to 0.40 wt%, a transition toward more refined and homogeneous surface morphology is observed, characterized by significant reduction in the size of dispersed natural rubber droplets (Figure 4e,f). This morphological refinement suggests that COE functions as an interfacial compatibilizer, lowering interfacial tension between the immiscible PHBV and NR phases. Such behavior aligns with findings by Seydibeyoğlu et al. [34] who demonstrated that functionalized vegetable oils enhance phase dispersion by acting as reactive bridges at polymer interfaces. Despite increased homogeneity, the surface displays uniformly distributed micro-voids. The presence of micro-voids indicates COE-rich phases within the PHBV/NR matrix; however, their limited size and even distribution suggest they originate from localized plasticizer-rich regions or microstructural rearrangements rather than macroscopic phase separation [36]. The high degree of uniformity and reduced surface heterogeneity at this loading level confirm that 0.40 wt% COE facilitates superior interfacial adhesion and improves overall apparent compatibility of the PHBV/NR/COE ternary system.

At higher COE loading of 0.75 wt%, surface morphology exhibits marked increase in pronounced and irregular void-like features (Figure 4g,h). The increased number of these features indicates excessive plasticization or saturation with COE at the adopted concentration, as reported in similar systems [19], with possible microphase separation that can disrupt PHBV matrix continuity and reduce structural uniformity. This observation suggests that beyond optimal COE concentration, excess plasticizer may compromise blend homogeneity and structural integrity.

## 4. Conclusions

This study evaluated the role of coffee oil epoxide (COE) as a bio-based plasticizer for PHBV/NR blends, with emphasis on thermal, mechanical, barrier, and exudation resistance properties. DSC analysis confirmed systematic T_g_ depression and reduced crystallinity with increasing COE content, indicating enhanced chain mobility. Mechanical testing further confirmed the plasticizing effect, with progressive reductions in tensile strength and modulus accompanied by substantial improvements in elongation at break, particularly at 0.75 wt% COE. WVTR decreased dramatically to ~0.67 g·m^−2^·h^−1^ for both 0.40% and 0.75% COE, suggesting a barrier plateau via hydrophobic tortuosity effects. Exudation studies revealed excellent plasticizer retention (0.21–0.53 wt% loss over 63 days), superior to conventional epoxidized oils. Morphological analysis using SEM revealed concentration-dependent surface evolution, with 0.40% COE exhibiting the most refined morphology characterized by significantly reduced rubber droplet size and improved phase homogeneity, confirming COE’s effectiveness as an interfacial compatibilizer. At 0.75% COE, increased irregular void-like features indicated excessive plasticization or potential microphase separation, consistent with the observed barrier property plateau. Results identify 0.40% COE as the optimal formulation, achieving maximum barrier improvement with balanced mechanical performance and efficient plasticizer utilization. The use of coffee waste-derived COE demonstrates the potential for waste valorization in bioplastic applications, converting post-consumption coffee residues into functional additives that enhance compatibility and flexibility in PHBV/NR blends.

Future work should investigate COE’s compatibility with other biopolymer blends, scale-up processing parameters for industrial applications, and comprehensive life cycle assessment to quantify the environmental benefits of these waste-derived formulations relative to conventional plasticizers. Additionally, comprehensive biodegradability studies would further establish the sustainability credentials of COE-plasticized PHBV/NR blends.

## Figures and Tables

**Table 1:** Sheet Extrusion Processing Parameters.

Processing Parameter	Value/Setting
Extruder type	Davis Standard DS20 (24:1 L/D)
Die Configuration	Coat-hanger die
Barrel Zone 1 Temperature	360 °F
Barrel Zone 2 Temperature	360 °F
Barrel Zone 3 Temperature	360 °F
Die Region Temperatures	360 °F
Die gap	0.38–0.42 mm
Screw speed	22 rpm

**Table 2:** Effect of Coffee oil epoxide (COE) Content on Thermal Properties of PHBV/NR (85:15) Blends.

Sample	ΔH_m_ (J/g)	T_m_ Onset (°C)	T_m_ Peak (°C) 1	T_m_ Peak (°C) 2	ΔH_c_ (J/g)	T_c_ Onset (°C)	T_c_ Peak (°C)	X_c_ (%)	T_g_ of PHBV	T_g_ of Rubber
PHBV/NR	79.6 ± 1.2	160.8 ± 0.4	165.4 ± 0.2	-	78.3 ± 1.4	120.1 ± 0.2	116.4 ± 0.2	54.5	1.8 ± 0.3	−62.6 ± 1.3
PHBV/NR + 0.30% COE	78.9 ± 1.0	160.7 ± 0.1	166.4 ± 0.2	-	77.3 ± 1.2	119.7 ± 0.1	115.9 ± 0.2	53.5	1.7 ± 0.3	−63.6 ± 1.4
PHBV/NR + 0.40% COE	76.8 ± 0.8	151.1 ± 0.8	158.9 ± 0.5	166.4 ± 0.2	68.7 ± 0.6	114.1 ± 0.6	109.1 ± 0.9	52.6	−4.3 ± 0.6	−65.6 ± 0.6
PHBV/NR + 0.75% COE	75.9 ± 1.8	149.8 ± 0.7	157.3 ± 0.5	165.5 ± 0.4	70.5 ± 1.6	113.4	108.4 ± 0.1	51.9	−5.6 ± 0.6	−64.6 ± 0.1

Note: Base formulation: 85 wt% PHBV, 15 wt% NR, 0.45 wt% peroxide, 0.63 wt% coagent; COE percentages based on total formulation weight. Background colors are used to distinguish melting-related, crystallization-related, and glass-transition parameters for different formulations. 1, 2: Due to the presence of bimodal melting peaks in the DSC thermograms, two melting temperatures (Tm_1_ and Tm_2_) are reported.

**Table 3:** Effect of COE Content on Mechanical Properties of PHBV/NR Blends.

Sample	Tensile Strength (MPa)	Elongation at Break (%)	Young’s Modulus (MPa)
PHBV	42.02 ± 3.14 ^a^	3.84 ± 1.07 ^ab^	2130.92 ± 163.99 ^a^
PHBV/NR	24.33 ± 1.15 ^c^	2.89 ± 0.35 ^b^	1421.28 ± 110.10 ^b^
PHBV/NR/0.3%COE	26.63 ± 1.10 ^b^	2.93 ± 0.25 ^b^	1538.79 ± 94.13 ^b^
PHBV/NR/0.4%COE	21.10 ± 1.58 ^d^	3.35 ± 0.75 ^ab^	1123.11 ± 73.11 ^c^
PHBV/NR/0.75%COE	20.39 ± 0.94 ^d^	3.97 ± 1.12 ^a^	1080.14 ± 62.95 ^c^

Note: Different letters denote statistically significant differences determined by one-way ANOVA (*p* < 0.05).

## Data Availability

The original contributions presented in the study are included in the article, further inquiries can be directed to the corresponding author.
